# Biochemical Comparison of *Anopheles gambiae* and Human NADPH P450 Reductases Reveals Different 2′-5′-ADP and FMN Binding Traits

**DOI:** 10.1371/journal.pone.0020574

**Published:** 2011-05-31

**Authors:** Lu-Yun Lian, Philip Widdowson, Lesley A. McLaughlin, Mark J. I. Paine

**Affiliations:** 1 School of Biological Sciences, University of Liverpool, Liverpool, United Kingdom; 2 Biomedical Research Institute, University of Dundee, Dundee, United Kingdom; 3 Liverpool School of Tropical Medicine, Liverpool, United Kingdom; Cinvestav, Mexico

## Abstract

NADPH-cytochrome P450 oxidoreductase (CPR) plays a central role in chemical detoxification and insecticide resistance in *Anopheles gambiae*, the major vector for malaria. *Anopheles gambiae* CPR (AgCPR) was initially expressed in *Eschericia coli* but failed to bind 2′, 5′-ADP Sepharose. To investigate this unusual trait, we expressed and purified a truncated histidine-tagged version for side-by-side comparisons with human CPR. Close functional similarities were found with respect to the steady state kinetics of cytochrome *c* reduction, with rates (*k*_ca*t*_) of 105 s^−1^ and 88 s^−1^, respectively, for mosquito and human CPR. However, the inhibitory effects of 2′,5′-ADP on activity were different; the IC_50_ value of AgCPR for 2′, 5′ –ADP was significantly higher (6–10 fold) than human CPR (hCPR) in both phosphate and phosphate-free buffer, indicative of a decrease in affinity for 2′, 5′- ADP. This was confirmed by isothermal titration calorimetry where binding of 2′,5′-ADP to AgCPR (*K*_d_ = 410±18 nM) was ∼10 fold weaker than human CPR (*K*_d_ = 38 nM). Characterisation of the individual AgFMN binding domain revealed much weaker binding of FMN (K_d_ = 83±2.0 nM) than the equivalent human domain (K_d_ = 23±0.9 nM). Furthermore, AgCPR was an order of magnitude more sensitive than hCPR to the reductase inhibitor diphenyliodonium chloride (IC_50_ = 28 µM±2 and 361±31 µM respectively). Taken together, these results reveal unusual biochemical differences between mosquito CPR and the human form in the binding of small molecules that may aid the development of ‘smart’ insecticides and synergists that selectively target mosquito CPR.

## Introduction

The mosquito *Anopheles gambiae* `is the principal vector for malaria in sub-Saharan Africa, a disease that affects over 500 million people worldwide. Insecticides have been the mainstay of disease control programmes in disease endemic countries for many years. However, these are threatened by the rapid evolution of insecticide resistance in disease vectors [1]. An important mechanism of resistance is enhanced metabolic inactivation of insecticides by P450s [2], a diverse superfamily of heme-containing monoxygenases that catalyse a diverse range of chemical reactions important in developmental processes and for detoxification of foreign compounds. It is well known that inhibiting P450 activity can help overcome resistance by potentiating insecticidal activity. Indeed piperonyl butoxide, a broad spectrum P450 inhibitor, is being increasingly used to extend the lifetime of pyrethroids, the only class of insecticide that can be safely used for insecticide treated bednets, in areas where resistance is undermining malaria control [3]. P450s are located in the endoplasmic reticulum where they require electrons supplied by NADPH cytochrome P450 oxidoreductase (CPR) for catalysis [4] placing this protein in a critical path in metabolism-based insecticide resistance, and a novel target for the development of new chemical synergists.

CPR is a ∼80 kDa microsomal diflavin reductase that contains flavin mononucleotide (FMN) and flavin adenenine dinucleotide (FAD) cofactors that shuttle electrons from the reduced form of NADPH through a series of redox-coupled reactions to P450 [4]; other physiological electron acceptors, include cytochrome *b*_5_, [5], [6], squalene hypoxidase [6] and heme oxygenase [7], [8]. The crystal structures of yeast [9], [10] and rat CPR have been resolved [11] revealing a structurally high conserved molecule with three separable domains: a short hydrophobic N-terminal membrane anchoring region (∼60 amino acids), essential for P450 coupling, followed by FMN-binding and FAD/NADPH-binding domains homologous to flavodoxin and ferrodoxin-NADP^+^ reductase respectively [11].

**Figure 1:**
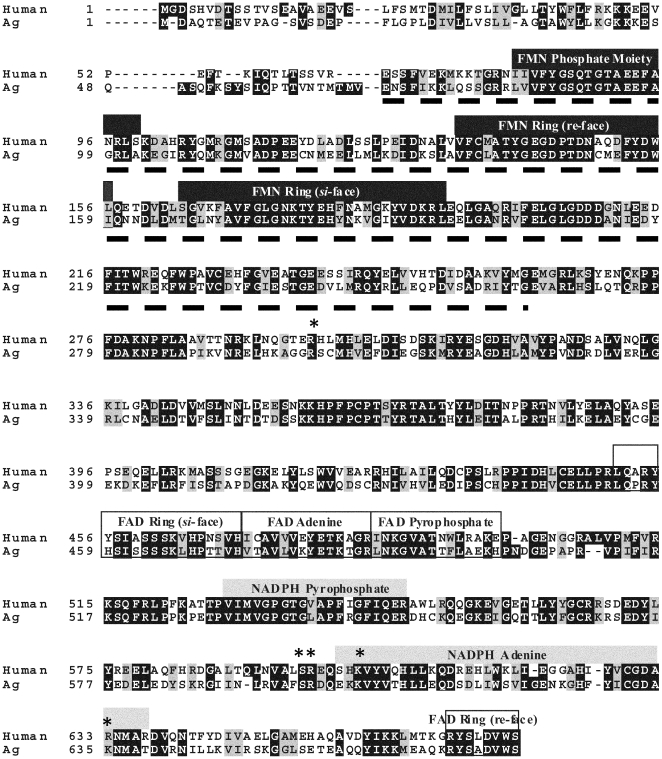
Sequence alignment of human and mosquito CPR. Cofactor binding sites are as defined by Wang *et al* [11]. Conserved residues associated with 2′-phosphate binding (S596, R597, K602 and R634 [17], [32]) are shown with an asterisk. The FMN binding domain spanning amino acids E69 to G254 that was expressed in *E. coli* is underlined.

Despite its great medical importance, there is relatively little known about the biochemistry and function of the individual components of the P450 complex of *A. gambiae*. Most recently, CPR from the mosquito *A. minimus* has been characterised [12], [13]. The mosquito enzyme shared close biochemical similarities with other CPR family members (Figure 1), although weak binding FMN and FAD cofactors [12] points to potential species differences. We have mapped the tissue distribution of CPR in *A. gambiae* and discovered high levels of expression in specialised mosquito cells (oenocytes) that suggests key physiological roles for CPR in metabolic processes and pheromone production/metabolism [14]. As expected, CPR gene knockdown by RNAi greatly increases the susceptibility of *A. gambiae* to permethrin, a widely used pyrethroid insecticide, emphasising the important chemoprotective role of the P450 monooxygenase complex in this organism [14], and validating its potential as a target for the development of chemical inhibitors that might enhance insecticidal activity.

Here we have expressed AgCPR and the equivalent human isoform in *E.coli* in order to allow direct comparisons of cofactor binding and steady-state kinetics. This has identified significant differences in the binding of 2′, 5′-ADP and FMN that suggest a potential target for the design of new insecticides or synergists to combat malaria.

## Materials and Methods

### Cloning, expression and purification of Δ63AgCPR

The cloning of AgCPR cDNA was previously described [14], [15]. The membrane anchor sequence was deleted by removal of amino acids 2–63 by PCR, using PFU polymerase (Stratagene) and the following oligonucleotides; forward primer: 5′- CGCG GAT CCG **ATG** ACG ATG ACG ATG GTG GAG ACC - 3′and reverse primer: 5′- TTC GGA TCC **TTA** GCT CCA CAC GTC CGC CGA – 3′ (*Bam*HI sites are underlined and the respective start and stop codons are indicated in bold). For molecular dissection of **Δ** 63AgCPR into its individual FMN- binding domain, PCR was used to amplify the amino acid residue region E69 to G254 using the following oligonucleotides: forward primer: 5′-CCATGGAGAACTCGTTCATCAAGAAGC – 3′ (NcoI site is underlined) and revese primers: 5′- CCATGGTTACTCGCCGGTGTAGATGC – 3′ (KpnI site underlined). The FMN –binding region expressed is underlined in Figure 1. The PCR product was digested with Kpn1 and Nco1 and subcloned into the expression vector pETM-11 which contained a TEV protease cleavage site. Constructs were confirmed by DNA sequencing. All proteins were expressed in *E. coli* strain BL21-CodonPlus(DE3)-RP strain at 30°C and purified using standard Ni^2+^-affinity chromatography and elution with 250 mM imidazole [16]. Thrombin (2000∶1(w/w) protein∶thrombin for AgCPR) and TEV protease (20∶1(w/w) protein∶TEV protease for the FMN binding domain) were, respectively, used to remove the His-tag from Δ63AgCPR. For thrombin cleavage, the sample was incubated for 2 hr at room temperature, whereas for TEV cleavage, overnight incubation at room temperature was required. The samples were reapplied to a Ni^2+^-affinity column to removed free his-tags and uncut protein. The samples were desalted in the presence of an excess of FMN and FAD and further purified using a 5 mL HiTrap Q FF anion exchange column (GE Healthcare) followed by MonoQ 5/5 anion exchange column (GE Healthcare). For final purification and buffer exchange, size exclusion chromatography was performed using a HiLoad 26/60 Superdex 75 column (50 mM Tris-HCl (Melford), 50 mM NaCl; pH 7.0). The human form, hCPR, was purified as described by Dohr [17]. Protein purity was >95% as assessed by SDS-PAGE and all samples were verified by mass spectrometry. Protein concentration was calculated by Bradford assay.

### Spectral Analysis

Absorption spectra of the purified CPR were carried out with a Cary 300 Bio and Cary 4000 spectrophotometers [18]. The sample buffer was 100 mM Tris, pH 8.0.

### Flavin content determination

Flavin cofactors (FMN and FAD) were released from AgCPR by heat denaturation (Paine et al, 1999). Briefly, 100 µL AgCPR (0.1 mg/mL) was incubated at 95°C for 5 min, centrifuged at room temperature at 20, 000×g for 5 min and the supernatant analysed for flavin content by HPLC analysis. 10 µL sample (or standards) were loaded into a mobile phase of 50 mM ammonium acetate; pH 4.5 with 20% (v/v) acetonitrile for separation on a 250 mm C_18_ column (Acclaim®120, Dionex) at 23°C. The flow-through was analysed by absorption spectroscopy and fluorescence (excitation 450 nm, emission 525 nm). Quantification was determined with reference to authentic FMN and FAD standards (Sigma).

#### Cytochrome *c* and NADPH kinetics

The rate of change of absorbance of horse heart cytochrome *c* (Sigma-Aldrich, UK) at 550 nm was measured at 25°C using a Cary 4000 UV-Visible spectrophotometer essentially as described [17]. For cytochrome *c* kinetics, 0.75 pmol purified AgCPR or hCPR was pre-incubated with 0–110 µM cytochrome *c* (dissolved in 0.3 M potassium phosphate buffer; pH 7.7 or 0.1 M Tris-HCl, pH 7.7/0.1 M KCl) in a total volume of 500 µl for 2 min at 25°C. Reactions were initiated by the addition of NADPH to a final concentration of 50 µM and rates measured in duplicate for 2 mins, a linear reaction range. For determination of NADPH kinetic parameters, the cytochrome *c* concentration was kept constant at 50 µM, and reactions were initiated with 0–150 µM NADPH.

### Inhibition measurement

#### Cytochrome *c*

Measurement of cytochrome *c* reduction was carried out at 25°C with 50 µM cytochrome *c* and 0.75 pmol purified AgCPR or hCPR essentially as described [17] using 0.3 M potassium phosphate buffer, pH 7.7 or 0.1 M Tris-HCl, pH 7.7/0.1 M KCl, and different concentrations of 2′, 5′-ADP, 2′-AMP, NADP or diphenyliodonium chloride (all from Sigma-Aldrich, UK). For phosphate buffer reactions AgCPR and hCPR reactions were initiated by the addition of 30 µM or 15 µM NADPH respectively, corresponding to their apparent K*_m_* values; for Tris buffer reactions respective NADPH concentrations were 12 µM and 5 µM.

### Isothermal Titration Calorimetry (ITC)

ITC experiments were performed using a ITC200 microcalorimeter (Microcal Inc/GE Healthcare). Protein samples were dialysed into 100 mM BES; pH 7.0 from 50% glycerol stocks stored at −20°C. The concentrations of all the proteins were determined by using Bradford assay. Samples were centrifuged at 13,000×g for 10 min and degassed at 23°C (2°C below the temperature at which the experiments were performed). All experiments were performed at 25°C. Typically, the protein concentration in the cell was 10–20 µM whilst ligand concentrations were in the range of 100–1000 µM. The data analysed using Origin 7.0 (Microcal) after subtracting the heats of dilution obtained from parallel experiments performed by injecting the nucleotide into the buffer. Thermodynamic parameters *n* (stoichiometry), *K*_d_ (1/*K*_a_, the association constant), and Δ°*H* (enthalpy change) were obtained by nonlinear least-squares fitting of experimental data using the single-site binding model. The free energy of binding (Δ*G*°) and entropy change (Δ*S*°) were obtained using eqs 1 and 2.

**Δ** *G° = −RT* ln *K*_A_ (1)

ΔG° = −*ΔH°*-*TΔS°* (2)

## Results

### Expression and purification of AgCPR and flavin-binding domains

**Figure 2:**
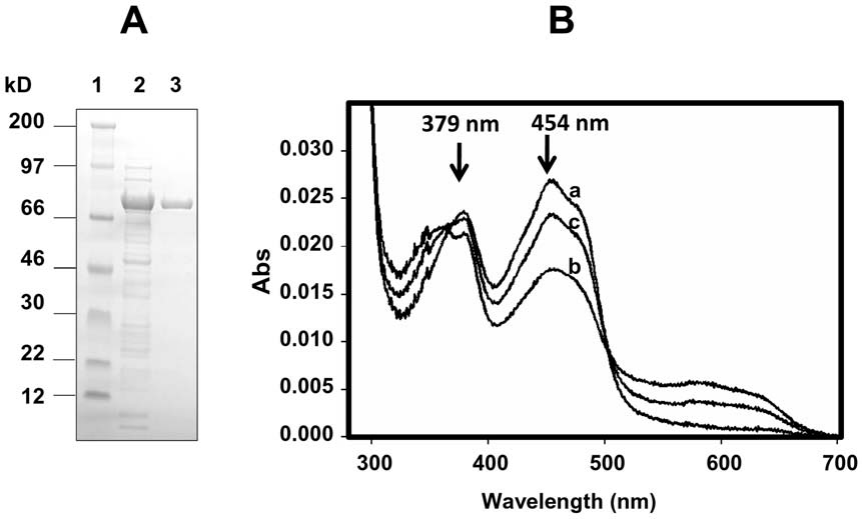
Purification a spectral characterisation of AgCPR. A, SDS-polyacrylamide gel electrophoresis of AgCPR purified by nickel-agarose affinity chromatography. Lane 1, molecular weight standards (kDa); Lane 2, partially purified CPR fraction eluted with 250 mM imidazole; Lane 3, thrombin cleaved AgCPR. B. absorption spectra of purified AgCPR(1.5 µM). Trace a is the oxidised spectrum; Trace b is the reduced spectrum with 1.5 µM NADPH and measured after 10 seconds; trace c is the air-stable semiquinone measured after 30 min.

Initial attempts to purify full-length AgCPR expressed in *E. coli* using standard ion exchange and 2, 5′-ADP-Sepharose affinity chromatography [19], [20] failed, which suggested that AgCPR might have different nucleotide binding properties. Therefore, to examine this further N-terminally histidine tagged soluble forms, lacking the N-terminal membrane anchor, of AgCPR was expressed in *E. coli* and affinity purified over nickel agarose (Fig. 2A). Although CPR lacking the amino-terminal membrane anchor loses its ability to interact with P450 it is otherwise fully functional and capable of reducing a range of electron acceptors [11]. Equivalent preparations of human anchorless CPR were also produced [17], and comparative enzyme activities measured via the reduction of cytochrome *c*, the surrogate electron acceptor most commonly used for measuring diflavin reductase activity [21].

Purified AgCPR was yellow, indicating the binding of flavin cofactors. AgCPR contained 0.72±0.01 mol of FMN and 0.80±0.01 mol of FAD per mol of enzyme. This compared with 0.88±0.03 mol of FMN and 0.92±0.02 mol of FAD per mol of hCPR enzyme purified under the same conditions, and somewhat higher than those for *A. minimus* (0.51 and 0.63 mol FMN and FAD per mol enzyme respectively) [12]. The optical spectrum of the oxidised enzyme was typical for a diflavin reductase with absorbance maxima at 379 nm and 454 nm (Fig. 2 B). The addition of a stoichiometric amount of NADPH produced a characteristic increase in the 500–650 nm region, associated with the formation of an air-stable flavin semiquinone as for human [22] and insect CPRs [12], [23]. The appearance of a peak in the 340–350 nm region in the semi-quinone form associated with NADPH oxidation similar to humans [24] was also evident.

### Comparison of Nucleotide Binding

**Figure 3:**
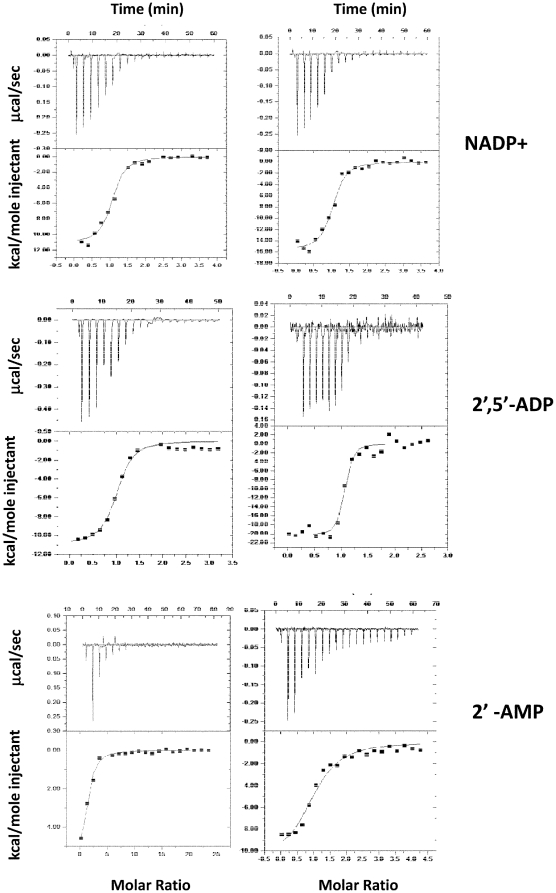
Isothermal titration of oxidized AgCPR with 2′, 5′ – ADP. Binding isotherms for the titration of 2′,5′-ADP into AgCPR. The points are fit to a one-set-of-sites model. Reactions were carried out in BES buffer (100 mM, pH 7.0 and 25°C) as described in Materials and Methods. The thermodynamic parameters of ΔS, ΔG, ΔH, and *K*_d_ are listed in Table 1.

Since AgCPR appeared to bind weakly to 2′, 5′-ADP the binding properties of this molecule were examined in more detail. 2′, 5′-ADP is the adenosine-ribose moiety of NADPH, which binds through a bi-partite mode with the nicotinamide moiety, to separate binding pockets of CPR [17]. Thus to delineate the roles played by the nicotinamide and ribose moieties, we used isothermal titration calorimetry (ITC) to determine the binding affinities of the adenosine-ribose fragments (Table 1). As previously reported for hCPR [25], this was done in a phosphate-free buffer to prevent competitive interactions with free phosphate. Figure 3 represents the ITC binding isotherms resulting from the titration of AgCPR with 2′, 5′-ADP, NADP+ and 2′-AMP. The binding isotherms were exothermic and fit to a single-site model. The observed dissociation binding constant for the AgCPR - 2′5′-ADP interaction was 410±18 nM, with a binding stoichiometry n = 1.03. This is substantially weaker than hCPR under the same reaction conditions (*K*_d_ of 38±3.8 nM; this study and [25]).

**Table 1:** Measured thermodynamic parameters for AgCPR and hCPR interactions with a selection of nucleotide ligands.

Ligand	*n* a	*K_obs_* (×10^5^ M^−1^)	*K_d_* (nM)^b^	Δ*H* (kcal mol^−1^)	*T*Δ*S* (kcal mol^−1^)	Δ*G* (kcal mol^−1^)
**AgCPR**						
**NADP^+^**	1.00±0.04	27.5±1.29	363±17	−11.97±0.64	−3.24	−8.73
**2′,5′-ADP**	1.01±0.03	24.4±1.07	410±18	−13.13±0.35	−4.73	−8.40
**2′-AMP**	1.01±0.22	2.51±0.11	4000±175	−7.66±1.98	−2.19	−5.47
**hCPR**						
**NADP^+^**	1.01±0.12	145±24.5	69±12	−19.46±2.92	−8.91	−10.55
**2′,5′-ADP**	1.00±0.02	263±26.3	38±3.8	−20.36±0.53	−9.97	−10.39
**2′-AMP**	1.02±0.06	6.15±1.69	1600±440	−11.07±0.88	−3.21	−7.86

Experiments were performed at 25°C in 100 mM BES; pH 7.0.

a Binding stoichiometry.

b *K*_d_ = 1/*K*_obs._

The binding affinity of NADP+ was ∼5-fold weaker for AgCPR (K_d_ = 363±17 nM) versus its human equivalent hCPR (K_d_ = 69±12 nM), while 2′-AMP binding was analysed to confirm the importance of the 2′-phosphate to nucleotide interaction with CPR. As expected the binding strength was orders of magnitude lower than 2′-5′-ADP and NADP+. Nevertheless a modest two-fold decrease in the apparent affinity for 2′ AMP in the mosquito protein (K_d_ of 4±0.18 µ M vs human 1.6±0.44 µ M), was measurable which compared to 2′5′-ADP suggest that interactions with the 5′ phosphate group make significant contributions to the binding affinity of the adenosine ribose moiety.

The enthalpy change (Δ*H*) for hCPR binding to 2′,5′-ADP was −20.36 kcal mol^−1^ was higher than for *A. gambiae* CPR binding (Δ*H* = −13.13 kcal mol^−1^) (Table 1). This is consistent with the lower dissociation constant value, and a further indication that the energetics of 2′,5′-ADP binding are more favourable with the human enzyme. Likewise, the Δ*H* for NADP^+^ binding to hCPR was approximately 2 fold lower than AgCPR (−19.46 kcal mol^−1^ vs −11.97 kcal mol^−1^). Finally, differences were minimal with respect to the energetics of 2′-AMP binding.

In summary, AgCPR binds all the nucleotide analogues more weakly than hCPR. Secondly, for both proteins, the small difference in affinities of each protein for NADP^+^ and 2′, 5′-ADP, suggests that in both the human and *Anopheles gambiae* proteins, the nicotinamide moiety makes only minimal contribution to the binding surface. From a structural viewpoint, it is likely that the conformations around the NADPH binding site in *Anopheles gambiae* CPR are similar to those already discovered for the mammalian proteins [10].

### Comparison of FMN Binding

**Figure 4:**
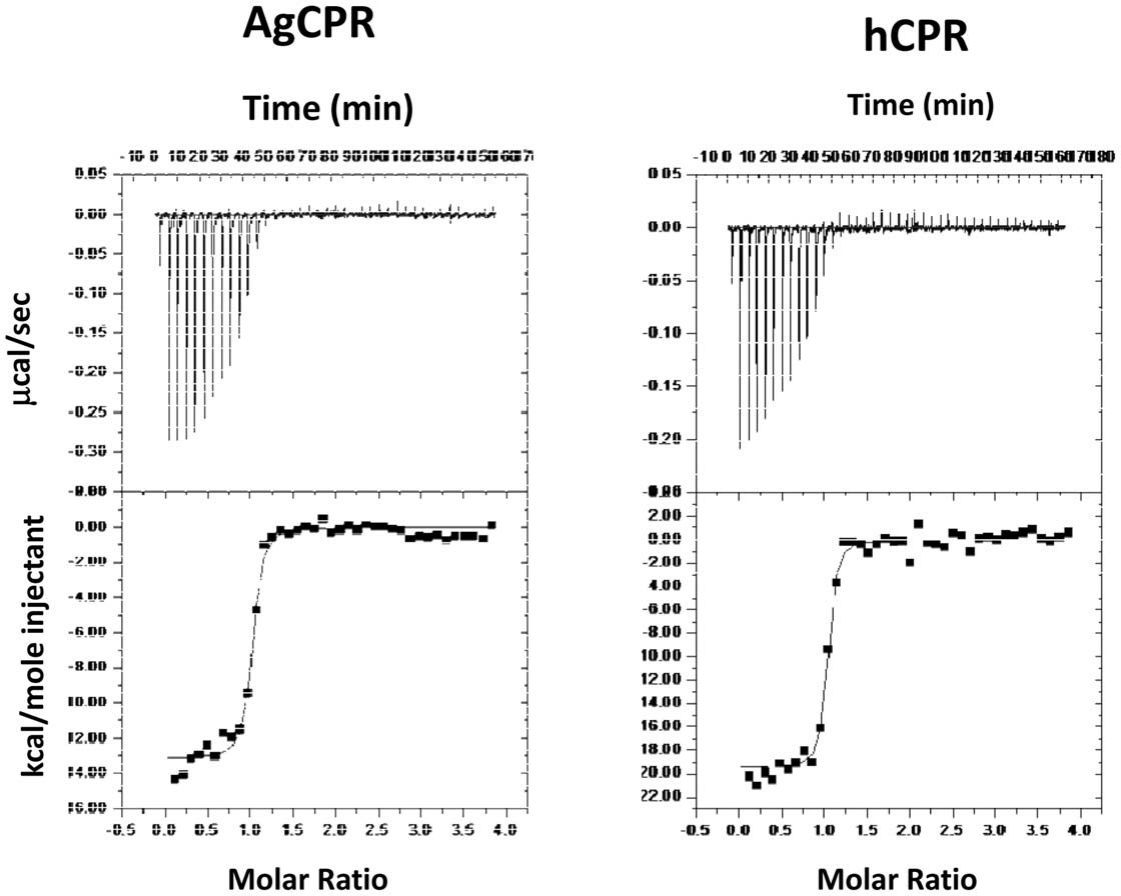
Isothermal titration of FMN domains with FMN. Binding isotherms for the titration of FMN into AgCPR. The points are fit to a one-set-of-sites model. Reaction were carried out in BES buffer (100 mM, pH 7.0 and 25°C) as described in Materials and Methods. The thermodynamic parameters of ΔS, ΔG, ΔH, and *K*_d_ are listed in Table 2.

The binding affinity of FMN to the isolated FMN-binding domains of both *A. gambiae* and hCPR were also examined by titration of the apo-FMN binding domains (Figure 4). Both isotherms show reasonable binding and saturation of the apo-proteins is achieved with excess flavin. The *K*_d_ for FMN binding to the isolated apo-human FMN-binding domain was calculated to be 23±0.9 nM which is almost 4-fold stronger than for the isolated apo-*A. gambiae* FMN-binding domain which was calculated to be 83±2.0 nM.

The thermodynamic characteristics (Table 2) also heavily favoured FMN binding to the human FMN-binding domain over the *A. gambiae* FMN-binding domain. The ΔH for FMN binding to the human FMN-binding domain was −19.46±0.35 kcal mol^−1^, which is more favourable than the −13.18±0.19 kcal mol^−1^ which was measured for the *A. gambiae* FMN-binding domain. The ΔG for FMN binding to the human FMN-binding domain was −10.28 kcal mol-1 suggesting the binding of FMN flavin to human FMN-binding domain is more favourable than to *A. gambiae* FMN-binding domain which had a calculated ΔG of −9.60 kcal mol−1.

**Table 2:** Measured thermodynamic parameters for *A. gambiae* and hCPR interaction with FMN.

apo-FMN domain	*n^a^*	*K_obs_* (×10^5^ M^−1^)	*K_d_* (nM)^b^	Δ*H* (kcal mol^−1^)	*T*Δ*S* (kcal mol^−1^)	Δ*G* (kcal mol^−1^)
**Human**	1.00±0.01	438±17.5	23±0.9	−19.46±0.35	−2.90	−10.28
***A. gambiae***	0.98±0.01	120±3.19	83±2.0	−13.18±0.19	−3.58	−9.60

Experiments were performed at 25°C in 100 mM BES.

### Kinetic comparisons between AgCPR and hCPR

To compare activities, standard phosphate buffer conditions that give optimal hCPR activity were initially used [17]. The specific activity of purified AgCPR was 23.9 µmol/mg/min compared with 18.3 µmol/mg/min for hCPR. Both AgCPR and hCPR catalysed NADPH-dependent cytochrome c reduction following Michaelis-Menton kinetics with respect to substrates cytochrome *c* and NADPH. The K*_m_* ^cytc^ values for human and mosquito enzymes were 19 µM and 23 µM respectively, indicating similar binding affinities for cytochrome *c*. There was a slight decrease in affinity of the mosquito CPR for the NADPH cofactor relative to the human protein (*K*_M_^NADPH^ 30.0 µM vs 16.0 µM respectively). Rates of cytochrome *c* reduction were alike, characterized by turnover numbers (*k*_ca*t*_) of 105 s^−1^ and 88 s^−1^ respectively for mosquito and hCPRs and near the range of 50–100 sec^−1^ noted for most CPRs [20]. Interestingly, the turnover rate for AgCPR is ∼4 fold higher than that reported for *A. minimus* CPR (23.8 s^−1^) [12], possibly attributable to the low stoichiometry of FMN in *A. minimus* CPR. Overall, however, close functional similarities were found with between human and *A. gambiae* CPR with respect to the steady state kinetics of cytochrome *c* reduction.

### Comparison of cytochrome *c* reductase inhibition by 2′, 5′-ADP

2′,5′-ADP, 2′-AMP and NADP+ were used as inhibitors of CPR catalyzed reduction of cytochrome *c* to determine whether the observed difference in ITC dissociation constants (*K*_d_) for complexes formed with 2′,5′-ADP were manifest as variations in inhibition (IC_50_) of catalytic activity (Table 3). Consistent with the lack of binding to 2′, 5′-ADP Sepharose, the major difference observed in phosphate buffer was a ten-fold decrease in affinity for 2′, 5′ - ADP, which inhibited AgCPR with an IC_50_ value of 262 µM compared with 28 µM for hCPR. There was also a two-fold increase in affinity for 2′ AMP (mosquito 468 µM vs human 1085 µM), while no significant differences were observed for NADP (mosquito 129 µM vs human 105 µM).

**Table 3:** Inhibition of cytochrome *c* reduction by adenosine molecules and diphenyliodonium chloride.

	IC_50_ µM (±S.E.)			
Compound	Phosphate Buffer		Tris Buffer^a^	
	Mosquito	Human	Mosquito	Human
2′, 5′-ADP	262±18	28±2	62±7	10±1
2′- AMP	1085±202	468±41	384±27	280±25
NADP	129±35	105±18	29±3	29±3
diphenyliodonium chloride	28±2	361±31	ND	ND

a ND = not done.

Since the binding affinity for NADPH is reduced in the presence of high phosphate concentrations [26], which may have influenced the data, we also investigated IC_50_ reactions in a phosphate-free Tris buffer (Table 3). Under these conditions we observed a ∼3 fold decrease in *K*_M_^NADPH^ values, relative to phosphate buffer, for human and mosquito enzymes (5.1 µM and 12.4 µM respectively), as well as corresponding decreases in *K_cat_* to 46.7 sec^−1^ and 57.5 sec^−1^ respectively, and a general decrease in IC_50_ values (Table 3). However, the 2′, 5′-ADP affinity for mosquito CPRs remained significantly lower (6 fold) than human. Differences in 2′-AMP and NADP IC_50_ values were negligible.

Finally, we also compared the inhibitory effects of diphenyliodonium chloride, a widely used inhibitor of CPR and related enzymes [27], on cytochrome *c* reductase activity. A difference of the same magnitude as that observed with 2′, 5′-ADP but in the opposite direction was also discovered: AgCPR is one order of magnitude more sensitive than hCPR to diphenyliodonium chloride (Table 3). Taken together, these results reveal significant biochemical differences between mosquito CPR and the human form in the binding of the small molecules 2′, 5′-ADP and diphenyliodonium chloride.

## Discussion

In view of the paucity of public health insecticides there is a pressing need to understand the mechanisms of detoxification and identify targets for new insecticides against disease vectors, in particular *A. gambiae.* Shutting down P450 activity is an attractive option since it can block resistance thereby extending the lifetime of insecticides already in use, as proven with the P450 inhibitor piperonyl butoxide[3]. Being the obligate redox partner for microsomal P450s, undermining Anopheline CPR activity is an attractive target provided it can be selectively inhibited. Although numerous CPRs have been characterised, few if any side-by-side comparisons have been made. Our direct comparative biochemical analysis of *A. gambiae* and human CPRs has revealed substantial differences in co-factor binding which may aid design of species specific CPR inhibitors. Of particular note was the weak affinity of AgCPR for 2′, 5′-ADP, reflected in the lack of binding to 2′, 5′-ADP affinity matrix and, ∼6–10 fold higher IC_50_ value compared to hCPR for cytochrome *c* reduction and ∼10 fold higher dissociation binding constant (*K*_d_ = 410 nM vs 38 nM for hCPR). This is significant since, for other CPRs, 2′,5′-ADP binding exerts conformational changes affecting electron transfer rates [25], [28], [29], while 2′-phosphate is the major contributor to the high affinity binding of NADPH to CPR [25], 25].

The results here also show that the *A. gambiae* protein binds the nucleotide analogues in ways similar to the human one (hCPR), with the nicotinamide moiety making only modest contributions to the binding surface. In the crystal structure of the rat reductase, NADP^+^ was found to bind in multiple conformations. It is, therefore, difficult to precisely identify the residues that might be important for nucleotide binding for the human protein which could provide clues as to why the binding affinities to Ag CPR are different. At this stage, we postulate that the considerably weaker binding of 2′,5′-ADP to AgCPR could have a significant influence on enzyme function

Differences also extend to FMN binding, where AgCPR again shows ∼4 fold weaker affinity against hCPR (*K*_d_ 23 nM vs 83 nM respectively). Interestingly, AgCPR is much more (∼10 fold) sensitive to diphenyliodonium chloride inhibition than hCPR. While there appears no obvious effect on cytochrome *c* reductase activity (the steady state kinetics AgCPR being comparable to hCPR), we have not compared activity with other redox partners such as P450s and heme oxygenase, where functional differences may be more pronounced. Indeed, given that heme degradation in mosquitoes is vital for survival with a blood feeding habit, and heme oxygenase is closely involved in heme degradation [30], it is feasible that biochemical differences in CPR may reflect differences in redox partner interactions. This hypothesis is supported by the fact that methionine synthase reductase, a diflavin reductase closely related to CPR, has a low affinity binding mode for 2′, 5′ –ADP (∼500 nM) [31].

An important question arising from this work is to what extent the observed differences in affinity of AgCPR for the adenosine, flavin and diphenyliodonium chloride molecules reflect intrinsic structural and/or mechanistic differences with respect to CPRs from human and other species. Since interactions involving 2′-phosphate are the major contributor to the high affinity binding of NADPH to CPR [26] we might expect differences in 2′, 5′-ADP to be associated with this phosphate molecule. However, the amino acids proposed to make up the 2′-phosphate binding motif including serine 596, arginine 597, lysine 602 and arginine 634 [17], [32] are conserved in *A. gambiae* CPR. Furthermore, these may prove difficult to resolve through X-ray crystallography due the inherent flexibility of this region in the CPR enzyme family [11].

The three-dimensional structures of the rat [11] and yeast [9] CPR and the rat nNOS FAD domain [33] have collectively provided details of the cofactor binding sites for the oxidoreductases. FMN binds in the region ^139^TYGEGPD and ^175^NKTYEHFN (rat CPR numbering), with Y140 and Y178 sandwiching the FMN isoalloxazine ring, and F181 stabilising the cofactor binding [34]. We have performed a multiple sequence alignment of AgCPR with *A. minimus* CPR, rat CPR, *S. cerevisiae* CPR and rat nNOS reductase domain (Fig. S1). As far as AgCPR is concerned, the residues mentioned above are all conserved with the exception of F181 (in rat CPR) replaced by Y184 (in AgCPR). However, it had previously been reported that mutation of F181 to Y181 in hCPR had minimal effects on FMN binding and enzymatic activity [34]; hence, replacing the tyrosine residue in AgCPR protein is of little consequence for FMN affinity. Therefore, despite the availability of good structures of homologous proteins, these have so far proved inadequate for understanding the cofactor binding characteristics of the AgCPR. Determination of the FMN-binding domain of AgCPR is in progress.

Finally, the greatly increased sensitivity of AgCPR for diphenyliodonium chloride may be related to increased rates of internal electron transfer, possibly linked to differences in redox potentials. Diphenyliodonium chloride inhibition is proposed to occur through an NADPH dependant reductive mechanism whereby diphenyliodonium chloride receives an electron from a reduced flavin to produce a reactive phenyl radical that recombines with FMN in its semiquinone state, creating an inactive phenylated FMN adduct [27].

### Conclusion

Overall, these results reveal significant biochemical differences between mosquito CPR and the human form in the binding of the small molecules 2′, 5′-ADP and diphenyliodonium chloride. Importantly, the low affinity of mosquito AgCPR for 2′, 5′-ADP and FMN and high affinity for diphenyliodonium chloride distinguishes it from human CPR, which offers the possibility of designing molecules to specifically inhibit mosquito CPR function. A potential advantage is that being encoded by a single gene that has a house-keeping role in furnishing electrons to all microsomal P450s, its inhibition is likely to be lethal to early development stages, giving it potential insecticidal properties in its own right. Notwithstanding the obvious challenges of designing molecules based on selective recognition of the NADPH binding pocket, these results suggest that detailed analysis of the structure and function of mosquito CPR may help in the development of ‘smart’ molecules that could specifically target mosquito vectors of major human diseases such as malaria. Finally, the importance of comparing AgCPR co-factor binding properties with other lentic invertebrate species must be emphasized as close similarities could limit the efficacy of inhibitors by collateral damage to non-target species.

## Supporting Information

Figure S1 **Multiple sequence alignments of the FMN binding region of** ***A. gambiae*** **CPR,** ***A. minimus*** **CPR, rat CPR (rCPR),** ***S. cerevisiae*** **CPR (yCPR) and the reductase domain of rat nNOS (nNOS).** Residues involved in FMN binding (light grey) and stabilisation (dark grey) are highlighted. Sequences were aligned using ClustalW.
(TIF)
